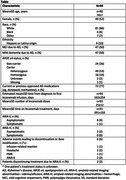# Real‐World Use of Lecanemab in Patients With Early Alzheimer’s Disease in the United States: a Case Series Review

**DOI:** 10.1002/alz70861_108599

**Published:** 2025-12-23

**Authors:** David C Weisman, Michael Henry Rosenbloom, Jose Soria‐Lopez, Gregory Cooper, Samuel Giles, Cara Leahy, Martin Sadowski, Curtis Schreiber, Paul E Schulz, Marwan N. Sabbagh, Christian J Camargo, Brooke Allen, Courtney Adams, Daryl Jones

**Affiliations:** ^1^ Abington Neurologic Associates, Abington, PA USA; ^2^ University of Washington Memory and Brain Wellness Center, Seattle, WA USA; ^3^ The Neuron Clinic, San Diego, CA USA; ^4^ Norton Neuroscience Institute, Louisville, KY USA; ^5^ Memory Treatment Centers, Jacksonville Beach, FL USA; ^6^ Memorial Healthcare Institute for Neuroscience, Owosso, MI USA; ^7^ New York University Langone Health, New York, NY USA; ^8^ Missouri Memory Center, Citizens Memorial Hospital, Bolivar, MO USA; ^9^ John P. and Kathrine G. McGovern Medical School at UTHealth, Houston, TX USA; ^10^ Barrow Neurological Institute, Phoenix, AZ USA; ^11^ University of Miami Miller School of Medicine, Miami, FL USA; ^12^ Roaring Fork Neurology, Basalt, CO USA; ^13^ Eisai Inc, Nutley, NJ USA; ^14^ Eisai Inc., Nutley, NJ USA

## Abstract

**Background:**

Lecanemab‐irmb (LEQEMBI®) is indicated for the treatment of patients with Alzheimer’s disease (AD) in the mild cognitive impairment (MCI) or mild dementia stage. While lecanemab’s efficacy and safety were proven in Phase 3 randomized controlled trials, real‐world data is important to understand its clinical outcomes beyond controlled trial settings. This study assessed real‐world utilization patterns of lecanemab and clinical outcomes of patients treated with lecanemab in the United States.

**Method:**

This multicenter, retrospective case series and patient pathway study was conducted in 15 geographically diverse neurology clinics, each abstracting deidentified medical chart data for up to 25 patients receiving lecanemab (≥7 infusions) and 1 neurologist per site completing an electronic survey plus an interview. This analysis examines the clinical characteristics, safety, and treatment outcomes of patients treated with lecanemab, including disease progression from baseline to last follow‐up visit. Descriptive statistics were run for the overall study population collected through April 11, 2025 (interim cutoff, ∼25% of total expected cases). The protocol received central institutional review board exemption.

**Result:**

At the interim analysis, 94 CRFs were completed (Table). The mean age of patients was 74 years, and 52% were female. Most patients were *APOE ε4* carriers (76%); 19% were homozygotes; 89% had no baseline microhemorrhages; 44% had baseline white matter hyperintensities. At the last follow‐up visit, 71% of patients had not progressed to the next disease state; 17% of these improved from mild AD dementia to MCI due to AD. Overall, 10 patients had ARIA events (8 were ARIA‐E and 2 were cerebral microhemorrhages or superficial siderosis [ARIA‐H]). Six patients experienced adverse events that resulted in discontinuation/dose modification of lecanemab therapy; of these, 2 were related to ARIA‐E.

**Conclusion:**

Overall, these findings underscore the benefits and safety profile of lecanemab in a real‐world clinical setting. The majority of patients did not progress to the next disease stage during the follow‐up period, with an average treatment duration exceeding a year (interim data). In a majority *APOE ε4* carrier population, 11% of patients experienced an ARIA event. The full data set analysis (data cutoff: May 23, 2025) will further explore these findings.